# Mitochondrial dynamic abnormalities in amyotrophic lateral sclerosis

**DOI:** 10.1186/s40035-015-0037-x

**Published:** 2015-07-29

**Authors:** Zhen Jiang, Wenzhang Wang, George Perry, Xiongwei Zhu, Xinglong Wang

**Affiliations:** Department of Pathology, Case Western Reserve University, Cleveland, OH USA; College of Sciences, University of Texas at San Antonio, San Antonio, TX USA

**Keywords:** ALS, Mitochondrial dysfunction, Mitochondrial dynamics, Mitochondrial fission and fusion, Mitochondrial trafficking, Mitochondrial biogenesis and mitophagy

## Abstract

Amyotrophic lateral sclerosis (ALS) is the most common motor neuron disease characterized by progressive loss of motor neurons in the brainstem and spinal cord. Currently, there is no cure or effective treatment for ALS and the cause of disease is unknown in the majority of ALS cases. Neuronal mitochondria dysfunction is one of the earliest features of ALS. Mitochondria are highly dynamic organelles that undergo continuous fission, fusion, trafficking and turnover, all of which contribute to the maintenance of mitochondrial function. Abnormal mitochondrial dynamics have been repeatedly reported in ALS and increasing evidence suggests altered mitochondrial dynamics as possible pathomechanisms underlying mitochondrial dysfunction in ALS. Here, we provide an overview of mitochondrial dysfunction and dynamic abnormalities observed in ALS, and discuss the possibility of targeting mitochondrial dynamics as a novel therapeutic approach for ALS.

## Introduction

Amyotrophic lateral sclerosis (ALS), also referred to as Lou Gehrig’s disease, typically develops between 50 and 60 years of age and progresses rapidly with the average survival of less than 30 months after diagnosis or onset [1]. ALS is the most common motor neuron disease characterized by progressive and fatal degeneration of both upper motor neurons in the motor cortex and lower motor neurons that connect the spinal cord and brainstem to muscle fibers [2], resulting in progressive muscle denervation, loss of motor function, muscle atrophy and eventual paralysis, speech deficit and finally death [3, 4]. Less than 10 % of ALS cases are familial (fALS), of which most are caused by repeat expansions of the C9ORF72 gene or mutations in genes encoding copper–zinc superoxide dismutase (SOD1), TAR DNA binding protein 43 (TDP-43) and fused in sarcoma (FUS). In contrast, 90–95 % of ALS cases, referred to as sporadic ALS (sALS), occur without any family history. The cellular and molecular mechanisms underlying motor neuron degeneration in both fALS and sALS are unknown, and effective treatments for this devastating and fatal disease are extremely limited.

Mitochondria are double membrane-bound organelles that are involved in multiple major cellular processes including ATP production, metabolite synthesis, calcium homeostasis, reactive oxygen species generation and even cell death [5, 6]. Due to limited glycolytic capacity, neurons particularly depend on mitochondria to maintain ion channel activities, synaptic transmission, and axonal/dendritic transport. In addition, as polarized cells with extended axons and dendrites, neurons require mitochondria to be efficiently transported and localized to sites with high metabolic and energy requirements [7]. Not surprisingly, a large number of studies suggest that mitochondria play a critical role in various major neurodegenerative diseases including ALS, Alzheimer’s disease, Parkinson’s disease and Huntington’s disease. Along this line, it was shown that SOD1 encoded by the first discovered gene associated with fALS, was localized to mitochondria [8], and involved in the regulation of mitochondrial function [9–13], underscoring the important role of mitochondria in ALS. In this review, we will focus on mitochondrial dynamic abnormalities in ALS and discuss mitochondrial dynamics as promising therapeutic targets.

### Mitochondrial dysfunction in ALS

Mitochondrial dysfunction has been consistently reported in ALS patients and ALS *in vitro* and *in vivo* experimental models, although the underlying molecular mechanism is still unclear. For instance, decreased activities of oxidative phosphorylation (OXPHOS) complexes I + III, II + III, IV, and citrate synthase were noticed in mitochondria from spinal cords of ALS patients [14, 15]. Consistently, the widely studied SOD1 G93A mouse model of ALS also demonstrated impaired activities of OXPHOS complexes I + III, II + III, IV [9]. Most importantly, mitochondrial dysfunction evidenced by reduced respiration and ATP synthesis precede rather than follow behavioral deficits, indicating an important role of mitochondrial dysfunction in disease progression [9]. Moreover, many ALS associated mutations in SOD1 result in the loss of antioxidant activity and the overproduction of reactive oxygen species (ROS) [16–19], and not surprisingly, a large number of studies reported increased oxidative stress or oxidative damage in spinal cords of ALS patients [20–22]. Elevated Ca^2+^ level in mitochondria was also reported in ALS patients [23] and ALS SOD1 transgenic mouse models [24–26], as as an early event preceding cytosolic Ca^2+^ increase and mutant SOD1 aggregation [27], further supporting the critical role of mitochondrial dysfunction in ALS pathogenesis.

### Mitochondrial morphology and fission/fusion dynamics in ALS

Although there is only one study showing abnormal mitochondrial outer membrane protrusions within axons of anterior root in ALS patients using biopsied tissues [28], abnormal mitochondrial morphology has been well documented in ALS experimental models. For example, previous studies from multiple groups showed that mitochondria became fragmented in cell and animal models expressing ALS-associated mutant SOD1 [29–33]. In addtion, we and other groups recently found that ALS-associated mutant TDP-43 overexpression also caused mitochondrial fragmentation in motor neurons *in vitro* and in mice [34–36, 33]. Studies in past decades reveal that mitochondria are highly dynamic organelles, and mitochondrial morphology results from the delicate balance of fission and fusion process [37, 38]. The mitochondrial fragmentation observed in ALS experimental models suggested a tipped balance of mitochondrial fission and fusion towards excessive fission due to increased fission, reduced fusion or both.

Mitochondrial fission and fusion processes are tightly regulated by several large dynamin-related GTPases that exert opposing effects [39]. Mitochondrial fission in mammals involves at least dynamin-like protein 1 (DLP1, also referred to as Drp1) and its recruiting factors on mitochondria such as Fis1, Mff, MiD49 and MiD51 [40]. On the other hand, mitochondrial fusion is governed by three large GTPase proteins: Mitofusin 1 (Mfn1), Mitofusin 2 (Mfn2) and optic atrophy protein 1(OPA1) [41]. Consistent with mitochondrial morphological changes, according to one recent study using SOD1 G93A transgenic mice, the protein levels of fission and fusion regulators including DLP1, Fis1, Mfn1 and OPA1 all increased before disease onset [42]. In contrast, during disease progression, the expression of Mfn1 and OPA1 but not DLP1 and Fis1 were found reduced. Altered expression of mitochondrial fission and fusion regulators such as DLP1 and Mfn1 were also reported in spinal cords of transgenic mice overexpressing wild type TDP-43 [34]. It still remains unknown how the changes in fission and fusion regulators correlate with and contribute to mitochondrial morphological alterations in SOD1 G93A and TDP-43 transgenic mice.

Aside from controlling mitochondrial morphology, mitochondrial fission and fusion dynamics are important for the maintenance of mitochondrial function [43]. Generally, when cells experience metabolic or environmental stresses, fusion enables the exchange of mitochondrial components within the mitochondrial network to compensate for damaged mitochondria, whereas fission helps to create new mitochondria to maintain a healthy mitochondria population [44]. On top of this, a recent study even reported that mitochondrial fission and fusion proteins regulate the assembly of respiratory complexes, indicating the direct involvement of mitochondrial fission and fusion dynamics in mitochondrial bioenergetics [45, 46]. Therefore, it is conceivable that the altered mitochondrial fission and fusion dynamics is likely a mechanism leading to mitochondrial dysfunction in ALS.

### Mitochondrial distribution and trafficking in ALS

In neurons, mitochondria are distributed strategically throughout the soma and axons to meet variant energy and metabolism requirements of different compartments. For example, mitochondria are usually found concentrated near synaptic terminals, where synaptic transmission and ion channel activity are highly energy demanding compared with other subcellular regions. However, remarkable mitochondrial accumulation was observed in the soma of motor neurons and proximal axon hillock region in the lumbar spinal cord of ALS patients [47]. Consistently, cultured motor neurons from SOD1 G93A transgenic mice demonstrated abnormal mitochondrial clusters in proximal axons [48]. SOD1 G93A transgenic rats also demonstrated accumulation of mitochondria clustered in axons of motor neurons [49]. Moreover, we and other groups reported altered mitochondrial distribution or mitochondrial aggregation around peri-nuclear area in motor neurons expressing ALS-associated TDP-43 mutant [36, 33, 50, 51].

Since mitochondrial distribution is closely regulated by mitochondrial transportation, one possible cause of abnormal mitochondrial distribution in ALS is altered mitochondrial trafficking, which is increasingly recognized as an important contributor in various neurodegenerative diseases [52, 53]. Mitochondria are transported bidirectionally in neurites along microtubules for fast movement and along actin filaments for slow movement via different motor-adaptor complexes [54]. Mitochondrial transportation is critical for newly generated mitochondria to move from the cell body to reach the distal segments of neurites, and for damaged mitochondria to move from distal neurite compartments to the cell body for degradation [55, 56]. Mitochondrial anterograde movement is mediated by kinesin motors whereas retrograde movement is regulated by dynein motors [57]. Kinesin and dynein motors are indirectly linked to mitochondria by Miro1-Milton adaptor complex [57]. Interestingly, our most recent study found that the expression of Miro 1, the only known mitochondrial outer membrane protein directly coupling mitochondria and motor-adaptor complexes, was significantly reduced in spinal cords of ALS patients, strongly suggesting impaired mitochondrial trafficking in ALS [58]. Consistently, the decreased expression of Miro1 was also noted in spinal cords but not brains of transgenic mice expressing ALS-associated SOD1 G93A or TDP-43 M337V mutant. In fact, we and other groups have provided evidence showing altered axonal transport of mitochondria in motor neurons expressing ALS-associated SOD1 mutant [59, 60] or TDP-43 mutant [36, 33]. Therefore, it is highly possible that Miro-1 deficiency is responsible for mitochondrial movement deficits in ALS and ALS experimental models. However, the possibility of direct interaction between SOD1 or TDP-43 and mitochondrial trafficking machinery can not be ruled out.

### Other mitochondrial dynamics in ALS

In addition to fission/fusion and movement, mitochondria function is also sensitive to changes in other mitochondrial dyanmics such as mitochondrial biogenesis and quality control (mitophagy) [61]. Mitochondria biogenesis is regulated by various factors, among which peroxisome proliferation activator receptor gamma-coactivator 1α (PGC-1α) has emerged as the master regulator. PGC-1α is a transcriptional coactivator that regulates the transcription of many genes including NRF1 and NRF2, which control the nuclear genes to encode mitochondrial protein, and TFAM, which drives transcription and replication of mtDNA [62]. It remains to be determined whether mitochondrial biogenesis is changed in ALS patients and experimental models. One study showed there is a loss of mitochondrial mass and reduced expression and activity of SIRT1, a regulator of PGC-1α, in neurons expressing SOD1 G93A mutant [63], suggesting the possible impairment of mitochondrial biogenesis. Damaged mitochondria are usually cleared by the process of mitophagy via mitochondrial quality control systems to maintain a healthy mitochondrial population within cells. The reduced expression of Parkin, an ubiquitin ligase implicated in mitophagy, was observed in transgenic mice expressing ALS associated mutant TDP-43 [64]. An ALS-associated mutation in Optineurin disrupts its function as a receptor for Parkin-mediated mitophagy [65]. In addition, other proteins such as valosin containing protein (VCP, or p97) or p62 were also reported in impairing mitophagy [66, 67]. Along this line, noteworthily, authophagy has been consistently implicated in neuronal loss in transgenic mice expressing ALS associated mutant SOD1 [68–70]. Interestingly, in addition to controlling mitochondrial morphology, previous studies demonstrated that mitochondrial fusion regulator Mfn2 was directly involved in the autophagosome formation [71] and the autophagosome-lysosome fusion [72]. Therefore, further studies might be interesting to test the interplay between autophagy and mitochondrial dynamics in the context of ALS.

### Mitochondrial dynamics as therapeutic targets of ALS

The widely used drug for ALS, i.e. riluzole, extends the life span of ALS patients by only three to six months [73, 74] highlighting the need for truly effective treatment options. Increasing evidence has revealed a prominent role for mitochondrial dysfunction in the pathogenesis of ALS and suggest mitochondria as promising therapeutic targets for ALS [75]. For example, SOD1 G93A mice administered CoQ10 in an effort to reduce oxidative stress and improve mitochondria function, demonstrated significantly increased survival [76]. Several chemicals specifically targeting mitochondria such as Olesoxime, Nortriptyline and Cyclosporine were reported as having neuroprotective effects in ALS cell and mouse models [77–80]. In fact, previous studies suggested that altering mitochondrial dynamics including fission/fusion, biogenesis and mitophagy might be viable therapeutic approaches for ALS. For instance, the inhibition of mitochondrial fission by the expression of DLP1 K38A, a dominant negative DLP1 mutant, was reported to prevent ALS-mutant SOD1 induced motor neuronal death [30]. Our recent study showed that the promotion of fusion by overexpression of Mfn2 significantly alleviated ALS-mutant TDP-43 induced mitochondrial and neuronal dysfunction in spinal cord motor neurons [36]. Moreover, resveratrol acting to promote mitochondrial biogenesis was found to significantly improve motor neuron function and extend the lifespan of SOD1 G93A mice [81, 63]. Finally, overexpression of the key biogenesis regulator PGC-1α could also alleviate ALS symptoms in SOD1 G37R transgenic mice [82]. Since mitochondrial function is sensitive to not only mitochondrial fission/fusion dynamics and biogenesis targeted by these strategies, it will be beneficial to investigate whether the manipulation of mitochondrial trafficking or mitophagy will also have some beneficial effect on mitochondria and neurons in ALS models.

## Conclusion

In addition to regulating mitochondrial morphology, mitochondrial fission and fusion are also involved in mitochondrial distribution and movement [83–85]. Further, changes in mitochondrial fission and fusion balance also affect mitophagy [44]. Moreover, PGC-1α was reported to affect mitochondrial morphology. Therefore, these different aspects of mitochondrial dynamics are not isolated but are in fact interrelated mechanisms. This may explain why almost all aspects of mitochondrial dynamics have been reported to be changed in ALS patients and/or ALS models. Notably, SOD1 and TDP-43, the most studied proteins associated with ALS, are found involved in the regulation of mitochondrial dynamics. While it still remains to be determined how altered mitochondrial dynamics contributes to the progression of ALS, like mitochondrial dysfunction, mitochondrial dynamic abnormalities appear to be early features of ALS, suggesting they play a critical role in the pathogenesis of this devastating disease. Supporting this notion, a most recent study showed that impaired mitochondrial trafficking through Miro1 deficiency specifically caused motor neuron degeneration and symptoms of motor neuron diseases [86]. The important role of mitochondrial dynamics in the pathogenesis of a wide range of neurological disorders including ALS, Alzheimer’s disease, Parkinson’s disease, brain ischemia and epilepsy has been increasingly recognized [87, 3, 88]. Therefore, it is likely that impaired mitochondrial dynamics might be a common mechanism leading to mitochondrial dysfunction and motor neuron degeneration in multiple forms of ALS.

## References

[1] Gordon PH (2013). Amyotrophic Lateral Sclerosis: An update for 2013 Clinical Features, Pathophysiology, Management and Therapeutic Trials. Aging and Dis.

[2] Leigh PN (2007). Chapter 13 Amyotrophic lateral sclerosis. Handb Clin Neurol.

[3] Su B, Wang X, Zheng L, Perry G, Smith MA, Zhu X (2010). Abnormal mitochondrial dynamics and neurodegenerative diseases. Biochim Biophys Acta.

[4] Tan W, Pasinelli P, Trotti D (2014). Role of mitochondria in mutant SOD1 linked amyotrophic lateral sclerosis. Biochim Biophys Acta.

[5] Benard G, Bellance N, James D, Parrone P, Fernandez H, Letellier T (2007). Mitochondrial bioenergetics and structural network organization. J Cell Sci.

[6] Delettre C, Lenaers G, Griffoin JM, Gigarel N, Lorenzo C, Belenguer P (2000). Nuclear gene OPA1, encoding a mitochondrial dynamin-related protein, is mutated in dominant optic atrophy. Nat Genet.

[7] Sheng ZH (2014). Mitochondrial trafficking and anchoring in neurons: New insight and implications. J Cell Biol.

[8] Kawamata H, Manfredi G (2010). Import, Maturation, and Function of SOD1 and Its Copper Chaperone CCS in the Mitochondrial Intermembrane Space. Antioxid Redox Sign.

[9] Mattiazzi M, D’Aurelio M, Gajewski CD, Martushova K, Kiaei M, Beal MF (2002). Mutated human SOD1 causes dysfunction of oxidative phosphorylation in mitochondria of transgenic mice. J Biol Chem.

[10] Liu J, Lillo C, Jonsson PA, Vande Velde C, Ward CM, Miller TM (2004). Toxicity of familial ALS-linked SOD1 mutants from selective recruitment to spinal mitochondria. Neuron.

[11] Bergemalm D, Jonsson PA, Graffmo KS, Andersen PM, Brannstrom T, Rehnmark A (2006). Overloading of stable and exclusion of unstable human superoxide dismutase-1 variants in mitochondria of murine amyotrophic lateral sclerosis models. J Neurosci.

[12] Deng HX, Shi Y, Furukawa Y, Zhai H, Fu RG, Liu ED (2006). Conversion to the amyotrophic lateral sclerosis phenotype is associated with intermolecular linked insoluble aggregates of SOD1 in mitochondria. P Natl Acad Sci USA.

[13] Israelson A, Arbel N, Da Cruz S, Ilieva H, Yamanaka K, Shoshan-Barmatz V (2010). Misfolded mutant SOD1 directly inhibits VDAC1 conductance in a mouse model of inherited ALS. Neuron.

[14] Wiedemann FR, Manfredi G, Mawrin C, Beal MF, Schon EA (2002). Mitochondrial DNA and respiratory chain function in spinal cords of ALS patients. J Neurochem.

[15] Borthwick GM, Johnson MA, Ince PG, Shaw PJ, Turnbull DM (1999). Mitochondrial enzyme activity in amyotrophic lateral sclerosis: implications for the role of mitochondria in neuronal cell death. Ann Neurol.

[16] Genova ML, Pich MM, Bernacchia A, Bianchi C, Biondi A, Bovina C (2004). The mitochondrial production of reactive oxygen species in relation to aging and pathology. Ann N Y Acad Sci.

[17] Adam-Vizi V (2005). Production of reactive oxygen species in brain mitochondria: contribution by electron transport chain and non-electron transport chain sources. Antioxid Redox Signal.

[18] Pitkanen S, Robinson BH (1996). Mitochondrial complex I deficiency leads to increased production of superoxide radicals and induction of superoxide dismutase. J Clin Invest.

[19] Rizzardini M, Mangolini A, Lupi M, Ubezio P, Bendotti C, Cantoni L (2005). Low levels of ALS-linked Cu/Zn superoxide dismutase increase the production of reactive oxygen species and cause mitochondrial damage and death in motor neuron-like cells. J Neurol Sci.

[20] Shaw PJ, Ince PG, Falkous G, Mantle D (1995). Oxidative damage to protein in sporadic motor neuron disease spinal cord. Ann Neurol.

[21] Ikawa M, Okazawa H, Tsujikawa T, Matsunaga A, Yamamura O, Mori T (2015). Increased oxidative stress is related to disease severity in the ALS motor cortex: A PET study. Neurology.

[22] Kihira T, Okamoto K, Yoshida S, Kondo T, Iwai K, Wada S (2013). Environmental characteristics and oxidative stress of inhabitants and patients with amyotrophic lateral sclerosis in a high-incidence area on the Kii Peninsula, Japan. Int Med.

[23] Siklos L, Engelhardt J, Harati Y, Smith RG, Joo F, Appel SH (1996). Ultrastructural evidence for altered calcium in motor nerve terminals in amyotropic lateral sclerosis. Ann Neurol.

[24] Damiano M, Starkov AA, Petri S, Kipiani K, Kiaei M, Mattiazzi M (2006). Neural mitochondrial Ca2+ capacity impairment precedes the onset of motor symptoms in G93A Cu/Zn-superoxide dismutase mutant mice. J Neurochem.

[25] Kim HJ, Magrane J, Starkov AA, Manfredi G (2012). The mitochondrial calcium regulator cyclophilin D is an essential component of oestrogen-mediated neuroprotection in amyotrophic lateral sclerosis. Brain: a J Neurol.

[26] Parone PA, Da Cruz S, Han JS, McAlonis-Downes M, Vetto AP, Lee SK (2013). Enhancing mitochondrial calcium buffering capacity reduces aggregation of misfolded SOD1 and motor neuron cell death without extending survival in mouse models of inherited amyotrophic lateral sclerosis. J Neurosci.

[27] Tradewell ML, Durham HD (2010). Calpastatin reduces toxicity of SOD1G93A in a culture model of amyotrophic lateral sclerosis. Neuroreport.

[28] Hirano A, Donnenfeld H, Sasaki S, Nakano I (1984). Fine structural observations of neurofilamentous changes in amyotrophic lateral sclerosis. J Neuropathol Exp Neurol.

[29] Vande Velde C, McDonald KK, Boukhedimi Y, McAlonis-Downes M, Lobsiger CS, Bel Hadj S (2011). Misfolded SOD1 associated with motor neuron mitochondria alters mitochondrial shape and distribution prior to clinical onset. PLoS One.

[30] Song W, Song Y, Kincaid B, Bossy B, Bossy-Wetzel E (2013). Mutant SOD1G93A triggers mitochondrial fragmentation in spinal cord motor neurons: neuroprotection by SIRT3 and PGC-1alpha. Neurobiol Dis.

[31] Vinsant S, Mansfield C, Jimenez-Moreno R, Del Gaizo MV, Yoshikawa M, Hampton TG (2013). Characterization of early pathogenesis in the SOD1(G93A) mouse model of ALS: part II, results and discussion. Brain and Behavior.

[32] Vinsant S, Mansfield C, Jimenez-Moreno R, Del Gaizo MV, Yoshikawa M, Hampton TG (2013). Characterization of early pathogenesis in the SOD1(G93A) mouse model of ALS: part I, background and methods. Brain and Behavior.

[33] Magrane J, Cortez C, Gan WB, Manfredi G (2014). Abnormal mitochondrial transport and morphology are common pathological denominators in SOD1 and TDP43 ALS mouse models. Hum Mol Genet.

[34] Xu YF, Gendron TF, Zhang YJ, Lin WL, D’Alton S, Sheng H (2010). Wild-type human TDP-43 expression causes TDP-43 phosphorylation, mitochondrial aggregation, motor deficits, and early mortality in transgenic mice. J Neurosci.

[35] Xu YF, Zhang YJ, Lin WL, Cao X, Stetler C, Dickson DW (2011). Expression of mutant TDP-43 induces neuronal dysfunction in transgenic mice. Mol Neurodegener.

[36] Wang W, Li L, Lin WL, Dickson DW, Petrucelli L, Zhang T (2013). The ALS disease-associated mutant TDP-43 impairs mitochondrial dynamics and function in motor neurons. Hum Mol Genet.

[37] Chan DC (2006). Mitochondria: dynamic organelles in disease, aging, and development. Cell.

[38] Bleazard W, McCaffery JM, King EJ, Bale S, Mozdy A, Tieu Q (1999). The dynamin-related GTPase Dnm1 regulates mitochondrial fission in yeast. Nat Cell Biol.

[39] McBride HM, Neuspiel M, Wasiak S (2006). Mitochondria: more than just a powerhouse. Current Biology : CB.

[40] Loson OC, Song Z, Chen H, Chan DC (2013). Fis1, Mff, MiD49 and MiD51 mediate Drp1 recruitment in mitochondrial fission. Mol Biol Cell.

[41] Detmer SA, Chan DC (2007). Functions and dysfunctions of mitochondrial dynamics. Nat Rev Mol Cell Biol.

[42] Liu W, Yamashita T, Tian F, Morimoto N, Ikeda Y, Deguchi K (2013). Mitochondrial fusion and fission proteins expression dynamically change in a murine model of amyotrophic lateral sclerosis. Curr Neurovasc Res.

[43] Picard M, Shirihai OS, Gentil BJ, Burelle Y (2013). Mitochondrial morphology transitions and functions: implications for retrograde signaling?. Am J Physiol Regul Integr Comp Physiol.

[44] Twig G, Elorza A, Molina AJ, Mohamed H, Wikstrom JD, Walzer G (2008). Fission and selective fusion govern mitochondrial segregation and elimination by autophagy. Embo J.

[45] Liu W, Acin-Perez R, Geghman KD, Manfredi G, Lu B, Li C (2011). Pink1 regulates the oxidative phosphorylation machinery via mitochondrial fission. Proc Natl Acad Sci U S A.

[46] Cogliati S, Frezza C, Soriano ME, Varanita T, Quintana-Cabrera R, Corrado M (2013). Mitochondrial cristae shape determines respiratory chain supercomplexes assembly and respiratory efficiency. Cell.

[47] Sasaki S, Iwata M (2007). Mitochondrial alterations in the spinal cord of patients with sporadic amyotrophic lateral sclerosis. J Neuropathol Exp Neurol.

[48] De Vos KJ, Chapman AL, Tennant ME, Manser C, Tudor EL, Lau KF (2007). Familial amyotrophic lateral sclerosis-linked SOD1 mutants perturb fast axonal transport to reduce axonal mitochondria content. Hum Mol Genet.

[49] Sotelo-Silveira JR, Lepanto P, Elizondo V, Horjales S, Palacios F, Martinez-Palma L (2009). Axonal mitochondrial clusters containing mutant SOD1 in transgenic models of ALS. Antioxid Redox Signal.

[50] Igaz LM, Kwong LK, Lee EB, Chen-Plotkin A, Swanson E, Unger T (2011). Dysregulation of the ALS-associated gene TDP-43 leads to neuronal death and degeneration in mice. J Clin Invest.

[51] Janssens J, Wils H, Kleinberger G, Joris G, Cuijt I, Ceuterick-de Groote C (2013). Overexpression of ALS-associated p. M337V human TDP-43 in mice worsens disease features compared to wild-type human TDP-43 mice. Mol Neurobiol.

[52] Guo X, Macleod GT, Wellington A, Hu F, Panchumarthi S, Schoenfield M (2005). The GTPase dMiro is required for axonal transport of mitochondria to Drosophila synapses. Neuron.

[53] Kieran D, Hafezparast M, Bohnert S, Dick JR, Martin J, Schiavo G (2005). A mutation in dynein rescues axonal transport defects and extends the life span of ALS mice. J Cell Biol.

[54] Frederick RL, Shaw JM (2007). Moving mitochondria: establishing distribution of an essential organelle. Traffic.

[55] Sasaki S, Iwata M (1996). Impairment of fast axonal transport in the proximal axons of anterior horn neurons in amyotrophic lateral sclerosis. Neurology.

[56] Silverstein B, Feld S, Kozlowski LT (1980). The availability of low-nicotine cigarettes as a cause of cigarette smoking among teenage females. J Health Soc Behav.

[57] Schwarz TL (2013). Mitochondrial trafficking in neurons. Cold Spring Harb Perspect Biol.

[58] Zhang F, Wang W, Siedlak SL, Liu Y, Liu J, Jiang K (2015). Miro1 deficiency in amyotrophic lateral sclerosis. Front Aging Neurosci.

[59] Ligon LA, LaMonte BH, Wallace KE, Weber N, Kalb RG, Holzbaur EL (2005). Mutant superoxide dismutase disrupts cytoplasmic dynein in motor neurons. Neuroreport.

[60] Wen HL, Lin YT, Ting CH, Lin-Chao S, Li H, Hsieh-Li HM (2010). Stathmin, a microtubule-destabilizing protein, is dysregulated in spinal muscular atrophy. Hum Mol Genet.

[61] Lim KL, Ng XH, Grace LG, Yao TP (2012). Mitochondrial dynamics and Parkinson’s disease: focus on parkin. Antioxid Redox Signal.

[62] Wu Z, Puigserver P, Andersson U, Zhang C, Adelmant G, Mootha V (1999). Mechanisms controlling mitochondrial biogenesis and respiration through the thermogenic coactivator PGC-1. Cell.

[63] Wang J, Zhang Y, Tang L, Zhang N, Fan D (2011). Protective effects of resveratrol through the up-regulation of SIRT1 expression in the mutant hSOD1-G93A-bearing motor neuron-like cell culture model of amyotrophic lateral sclerosis. Neurosci Lett.

[64] Stribl C, Samara A, Trumbach D, Peis R, Neumann M, Fuchs H (2014). Mitochondrial dysfunction and decrease in body weight of a transgenic knock-in mouse model for TDP-43. J Biol Chem.

[65] Wong YC, Holzbaur EL (2014). Optineurin is an autophagy receptor for damaged mitochondria in parkin-mediated mitophagy that is disrupted by an ALS-linked mutation. Proc Natl Acad Sci U S A.

[66] Kimura Y, Fukushi J, Hori S, Matsuda N, Okatsu K, Kakiyama Y (2013). Different dynamic movements of wild-type and pathogenic VCPs and their cofactors to damaged mitochondria in a Parkin-mediated mitochondrial quality control system. Genes Cells.

[67] Gal J, Strom AL, Kwinter DM, Kilty R, Zhang J, Shi P (2009). Sequestosome 1/p62 links familial ALS mutant SOD1 to LC3 via an ubiquitin-independent mechanism. J Neurochem.

[68] Li L, Zhang X, Le W (2008). Altered macroautophagy in the spinal cord of SOD1 mutant mice. Autophagy.

[69] Zhang XJ, Li LA, Chen S, Yang DH, Wang Y, Zhang X (2011). Rapamycin treatment augments motor neuron degeneration in SOD1(G93A) mouse model of amyotrophic lateral sclerosis. Autophagy.

[70] Zhang X, Chen S, Song L, Tang Y, Shen Y, Jia L (2014). MTOR-independent, autophagic enhancer trehalose prolongs motor neuron survival and ameliorates the autophagic flux defect in a mouse model of amyotrophic lateral sclerosis. Autophagy.

[71] Hailey DW, Rambold AS, Satpute-Krishnan P, Mitra K, Sougrat R, Kim PK (2010). Mitochondria supply membranes for autophagosome biogenesis during starvation. Cell.

[72] Zhao T, Huang X, Han L, Wang X, Cheng H, Zhao Y (2012). Central role of mitofusin 2 in autophagosome-lysosome fusion in cardiomyocytes. J Biol Chem.

[73] Bensimon G, Lacomblez L, Meininger V (1994). A controlled trial of riluzole in amyotrophic lateral sclerosis. ALS/Riluzole Study Group. N Engl J Med.

[74] Lacomblez L, Bensimon G, Leigh PN, Guillet P, Meininger V (1996). Dose-ranging study of riluzole in amyotrophic lateral sclerosis. Amyotrophic Lateral Sclerosis/Riluzole Study Group II. Lancet.

[75] Cozzolino M, Carri MT (2012). Mitochondrial dysfunction in ALS. Prog Neurobiol.

[76] Matthews RT, Yang L, Browne S, Baik M, Beal MF (1998). Coenzyme Q10 administration increases brain mitochondrial concentrations and exerts neuroprotective effects. Proc Natl Acad Sci U S A.

[77] Bordet T, Buisson B, Michaud M, Drouot C, Galea P, Delaage P (2007). Identification and characterization of cholest-4-en-3-one, oxime (TRO19622), a novel drug candidate for amyotrophic lateral sclerosis. J Pharmacol Exp Ther.

[78] Martin LJ (2010). Olesoxime, a cholesterol-like neuroprotectant for the potential treatment of amyotrophic lateral sclerosis. IDrugs: The Invest Drugs J.

[79] Wang H, Guan Y, Wang X, Smith K, Cormier K, Zhu S (2007). Nortriptyline delays disease onset in models of chronic neurodegeneration. Eur J Neurosci.

[80] Keep M, Elmer E, Fong KS, Csiszar K (2001). Intrathecal cyclosporin prolongs survival of late-stage ALS mice. Brain Res.

[81] Price NL, Gomes AP, Ling AJ, Duarte FV, Martin-Montalvo A, North BJ (2012). SIRT1 is required for AMPK activation and the beneficial effects of resveratrol on mitochondrial function. Cell Metab.

[82] Da Cruz S, Parone PA, Lopes VS, Lillo C, McAlonis-Downes M, Lee SK (2012). Elevated PGC-1alpha activity sustains mitochondrial biogenesis and muscle function without extending survival in a mouse model of inherited ALS. Cell Metab.

[83] Li Z, Okamoto K, Hayashi Y, Sheng M (2004). The importance of dendritic mitochondria in the morphogenesis and plasticity of spines and synapses. Cell.

[84] Misko A, Jiang S, Wegorzewska I, Milbrandt J, Baloh RH (2010). Mitofusin 2 is necessary for transport of axonal mitochondria and interacts with the Miro/Milton complex. J Neurosci.

[85] Wang X, Su B, Lee HG, Li X, Perry G, Smith MA (2009). Impaired balance of mitochondrial fission and fusion in Alzheimer’s disease. J Neurosci.

[86] Nguyen TT, Oh SS, Weaver D, Lewandowska A, Maxfield D, Schuler MH (2014). Loss of Miro1-directed mitochondrial movement results in a novel murine model for neuron disease. Proc Natl Acad Sci U S A.

[87] Shi P, Gal J, Kwinter DM, Liu X, Zhu H (2010). Mitochondrial dysfunction in amyotrophic lateral sclerosis. Biochim Biophys Acta.

[88] Archer SL (2013). Mitochondrial dynamics--mitochondrial fission and fusion in human diseases. N Engl J Med.

